# Supplementary material for: SEA CDM: Study-Experiment-Assay Common Data Model and Databases for Cross-Domain Data Integration and Analysis
Source: Sci Data. 2026 Jan 14;13:238. doi: 10.1038/s41597-026-06558-z (PMC12905146; doi:10.1038/s41597-026-06558-z)
Supplement: Supplementary file 1 — Supplemental Figures [file 41597_2026_6558_MOESM1_ESM.pdf]

Supplemental Figures.

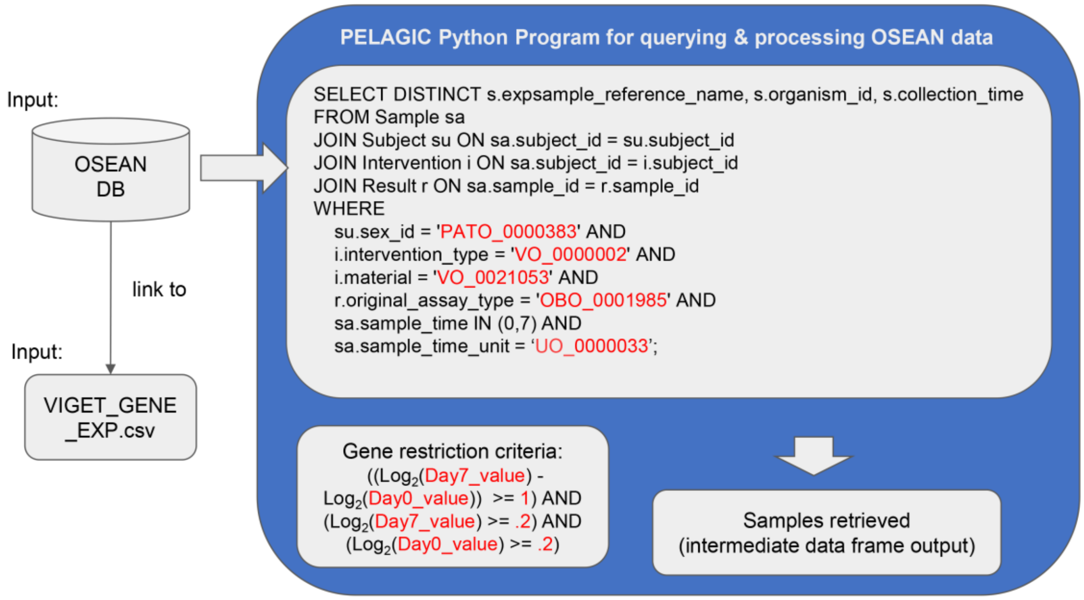

Supplemental Figure 1. Alternative Ontology-based Query for VIGET OSEAN database. This figure provides an alternative query using the ontology IDs instead of the main queries as part of **Figure 4**. These queries are identical due to prior data harmonization ensuring that each attribute pair (name, ontology_id) is a 1:1 match. The time units were not included as part of the Figure 4 for legibility. The mapping for each figure and ontology ID is as follows: 'PATO_0000383' is 'female'; 'VO_0000002' is 'vaccination'; 'VO_0021053' is FluMist; 'OBI_0001985' is 'microarray assay'; and 'UO_0000033' is 'day'.

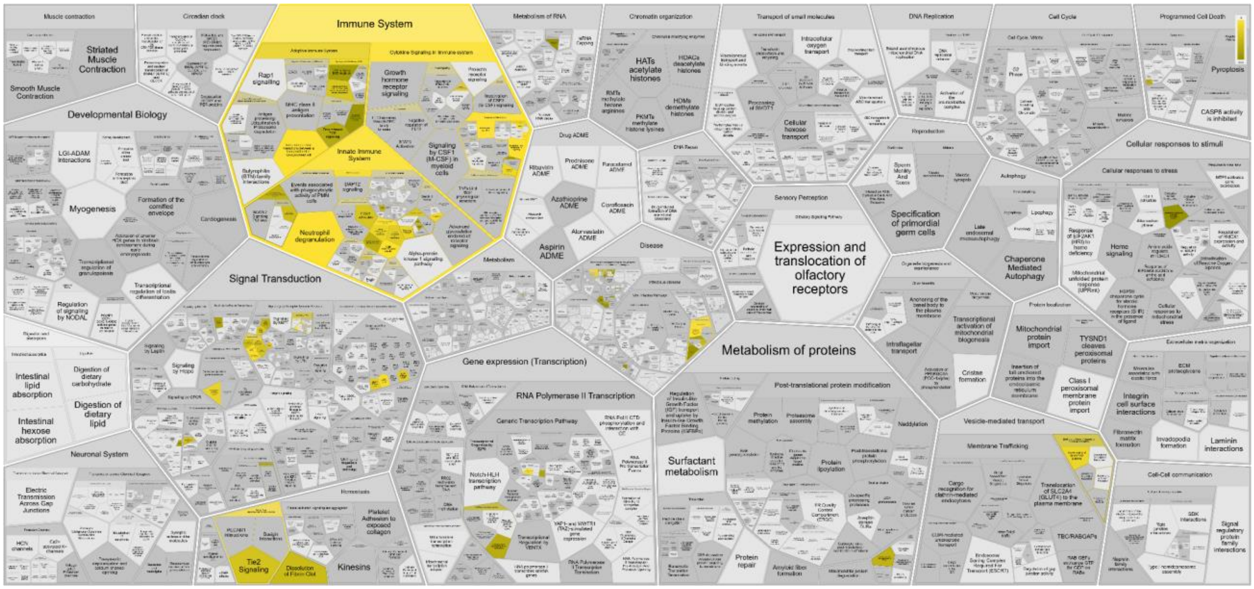

Supplemental Figure 2. Full Reacfome representation of Reactome pathways stimulated by all Influenza vaccines in all-sex human subjects. Gene set enrichment values can be found as part of Supplemental File 3.

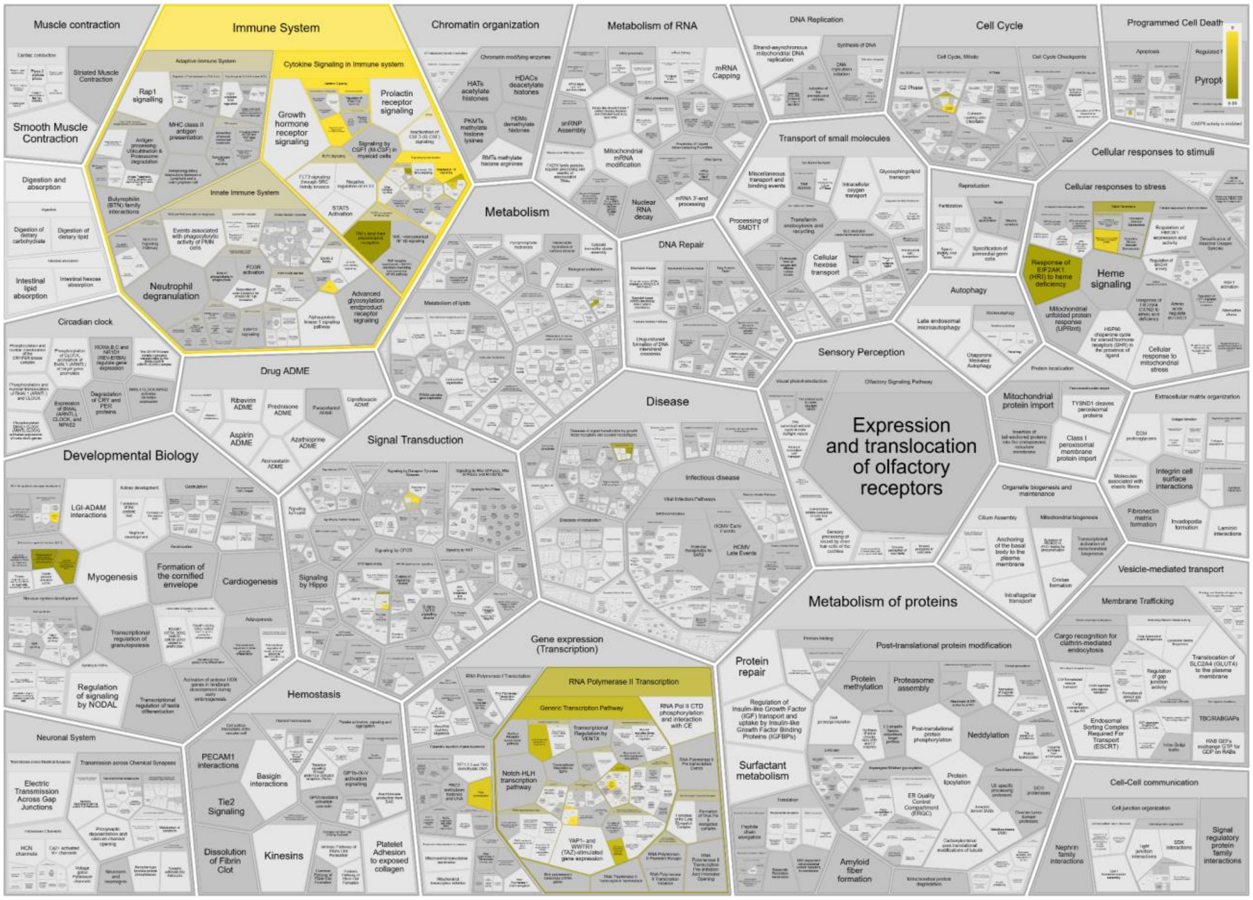

Supplemental Figure 3. Full Reacfome representation of Reactome pathways stimulated by all Influenza vaccines in female human subjects. Gene set enrichment values can be found as part of Supplemental File 3.

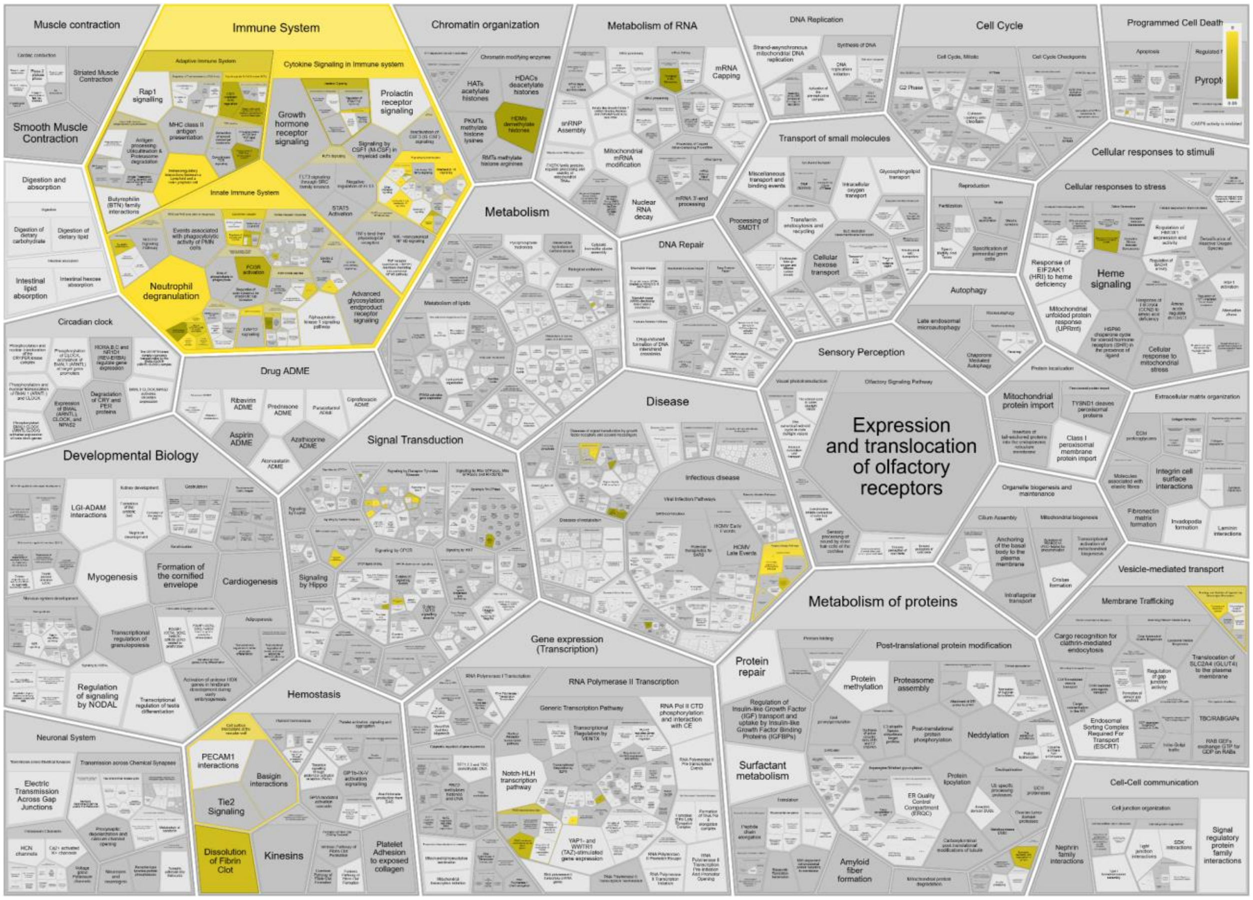

Supplemental Figure 4. Full Reacfome representation of Reactome pathways stimulated by all Influenza vaccines in male human subjects. Gene set enrichment values can be found as part of Supplemental File 3.

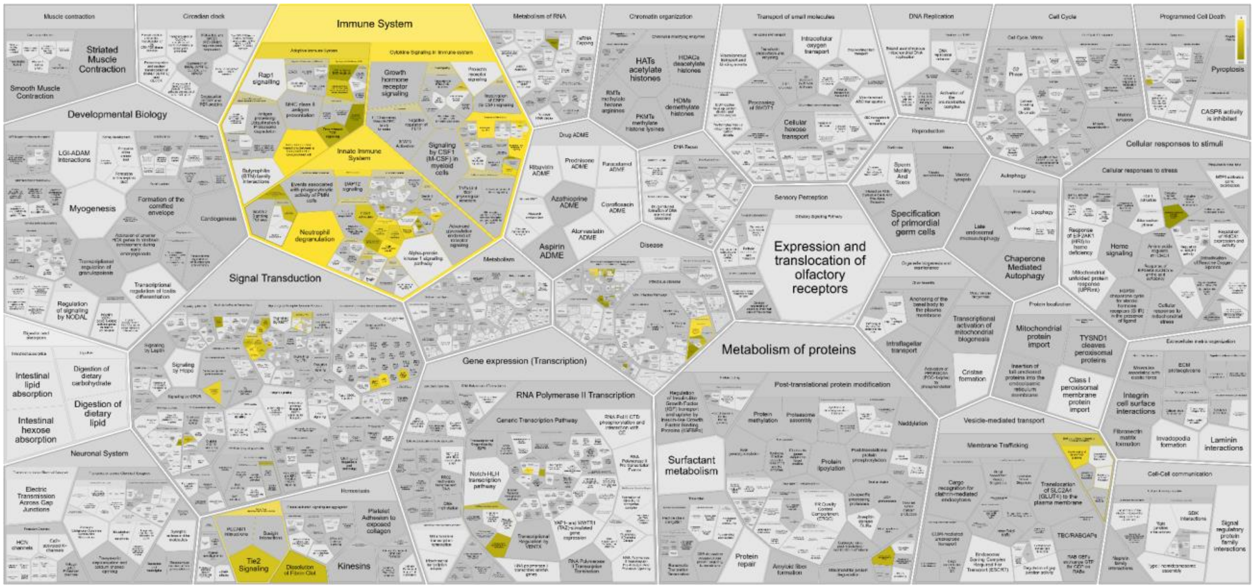

Supplemental Figure 5. Full Reacfome representation of Reactome pathways stimulated by live attenuated Influenza vaccines in all sexes. Gene set enrichment values can be found as part of Supplemental File 3.

-

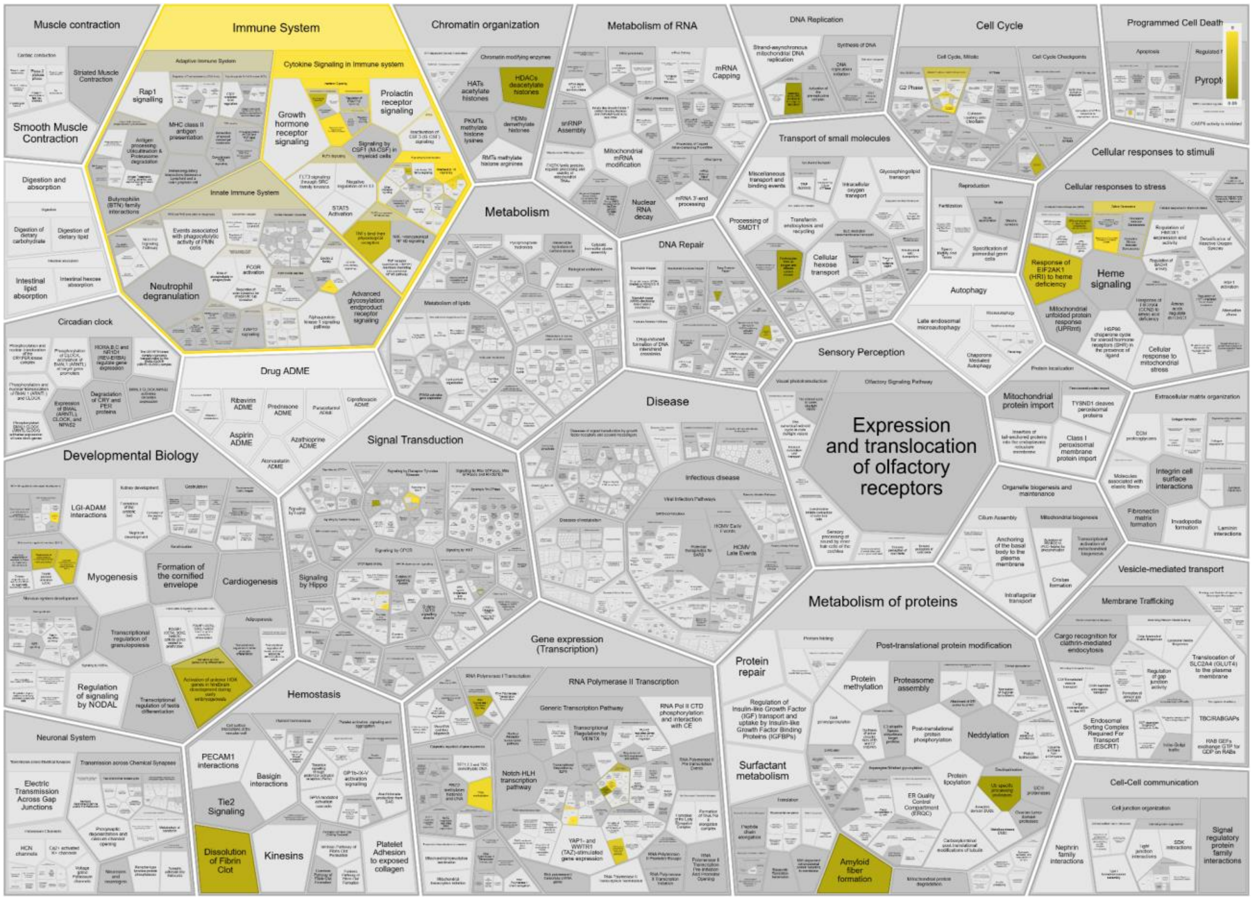

Supplemental Figure 6. Full Reacfome representation of Reactome pathways stimulated by all Influenza and live attenuated Influenza vaccines in female human subjects. Gene set enrichment values can be found as part of Supplemental File 3.

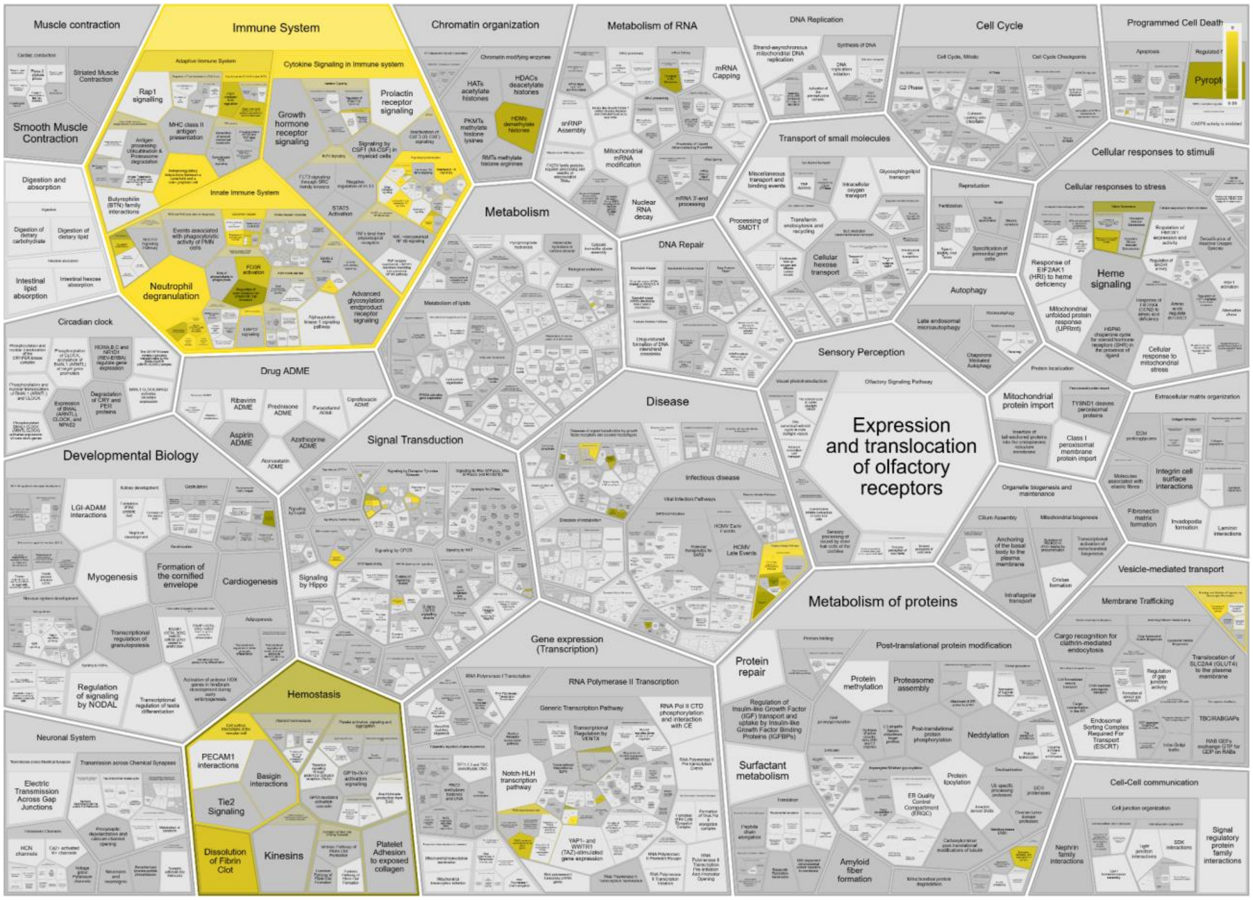

Supplemental Figure 7. Full Reacfome representation of Reactome pathways stimulated by live attenuated Influenza vaccines in male human subjects. Gene set enrichment values can be found as part of Supplemental File 3.

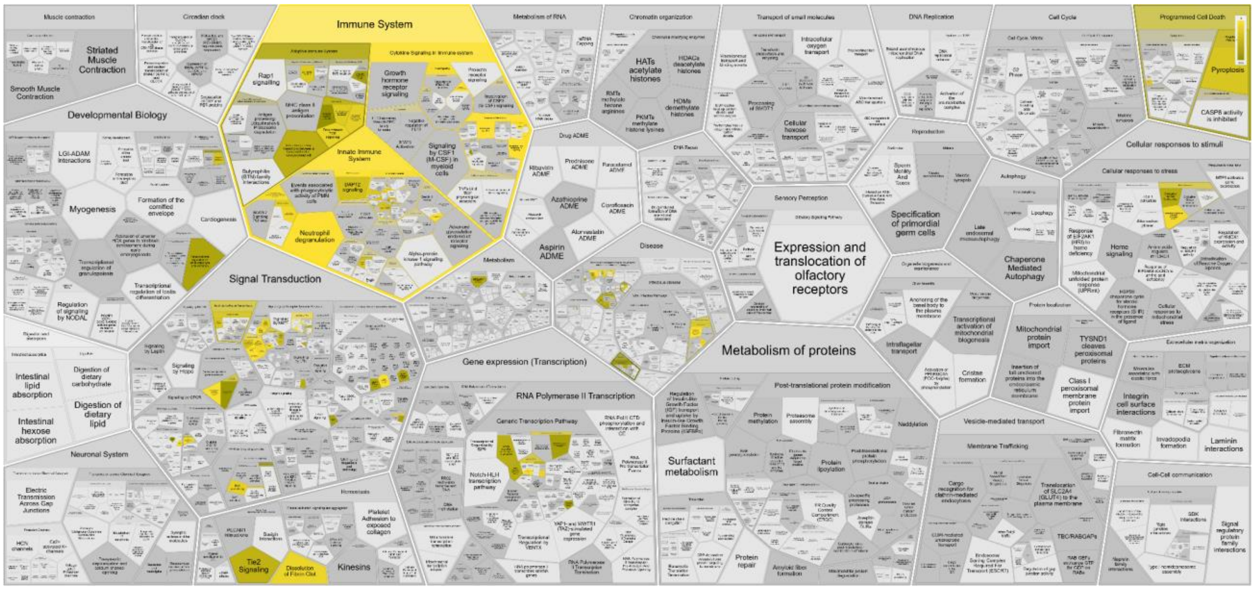

Supplemental Figure 8. Full Reacfome representation of Reactome pathways stimulated by trivalent inactivated Influenza vaccines in all-sexes. Gene set enrichment values can be found as part of Supplemental File 3.

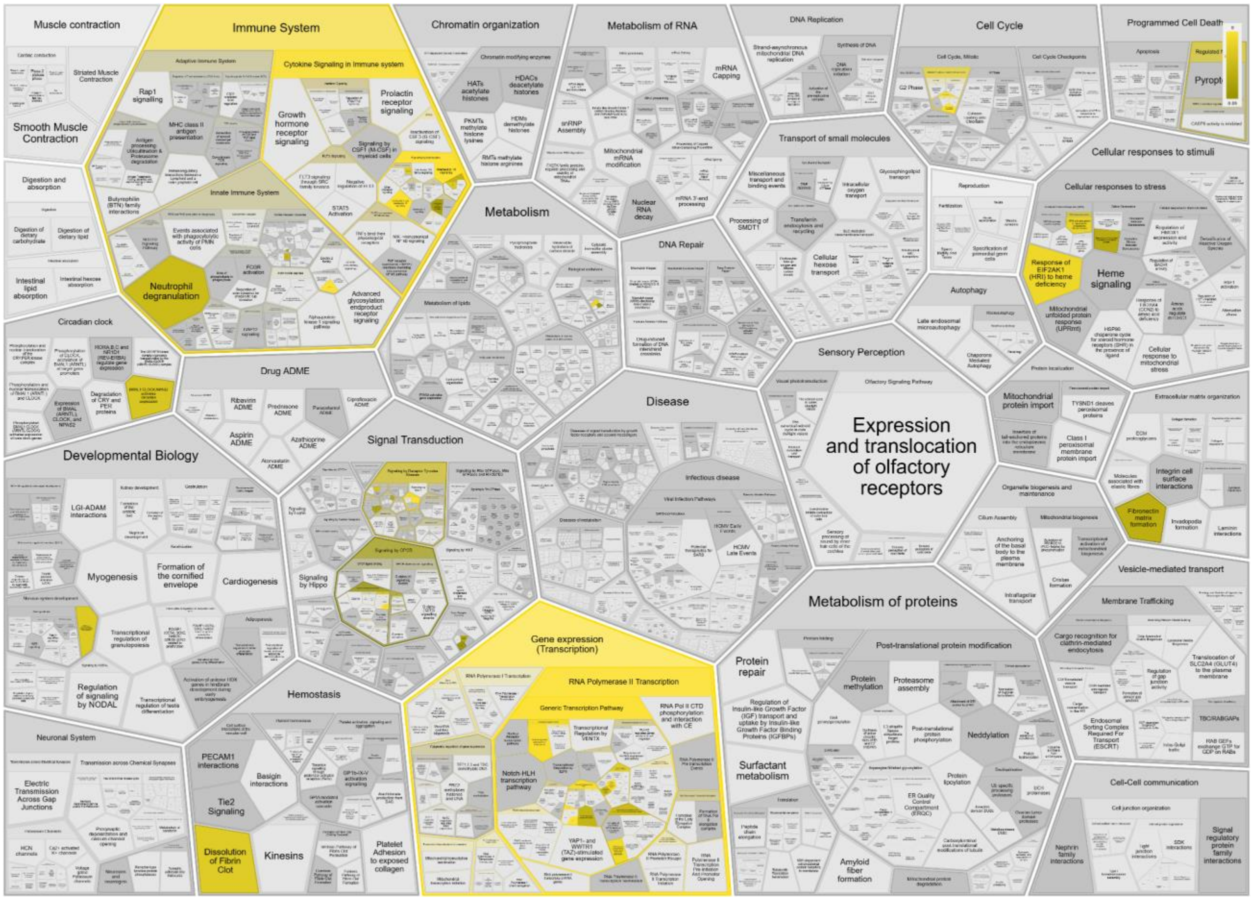

Supplemental Figure 9. Full Reacfome representation of Reactome pathways stimulated by trivalent inactivated Influenza vaccines in female human subjects. Gene set enrichment values can be found as part of Supplemental File 3.

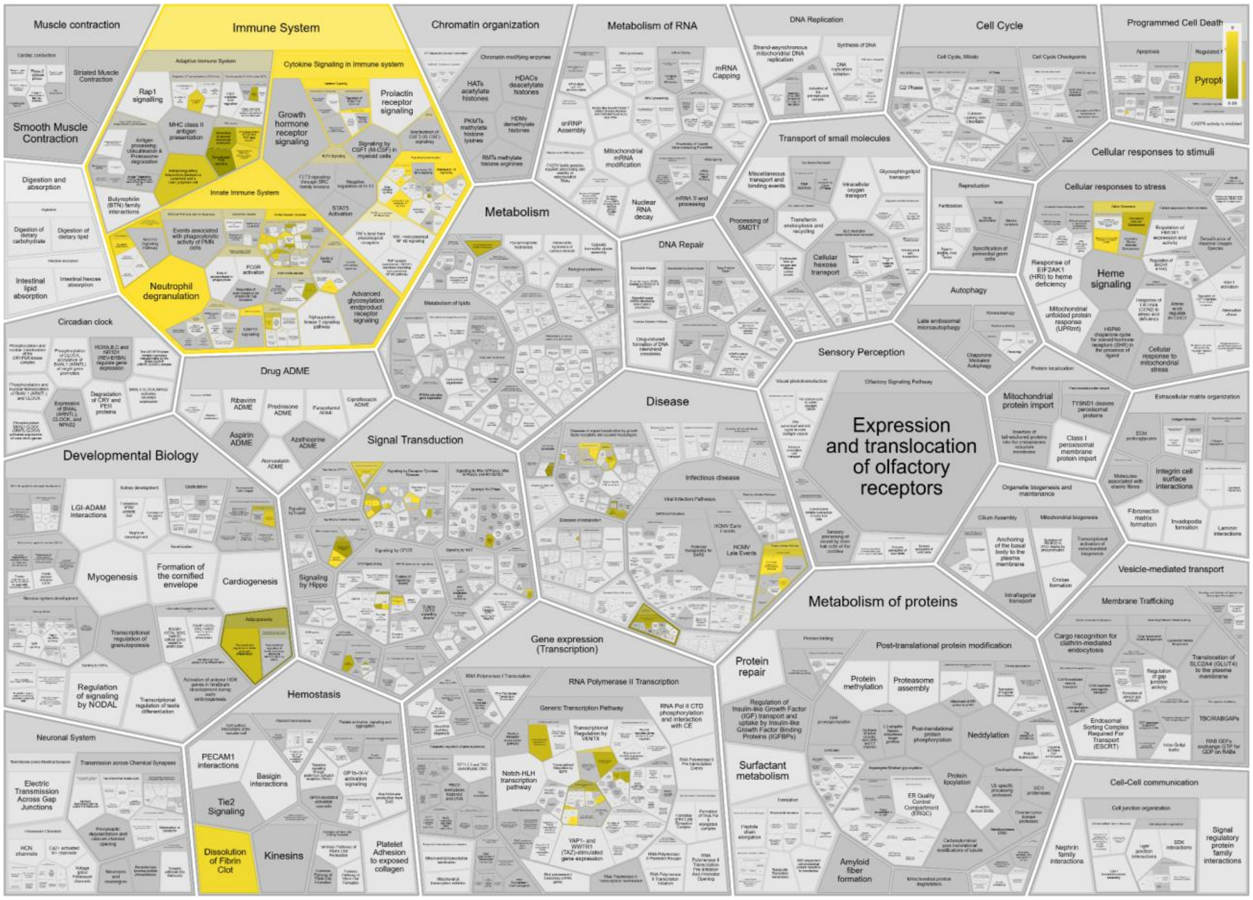

Supplemental Figure 10. Full Reacfome representation of Reactome pathways stimulated by trivalent inactivated Influenza vaccines in male human subjects. Gene set enrichment values can be found as part of Supplemental File 3.

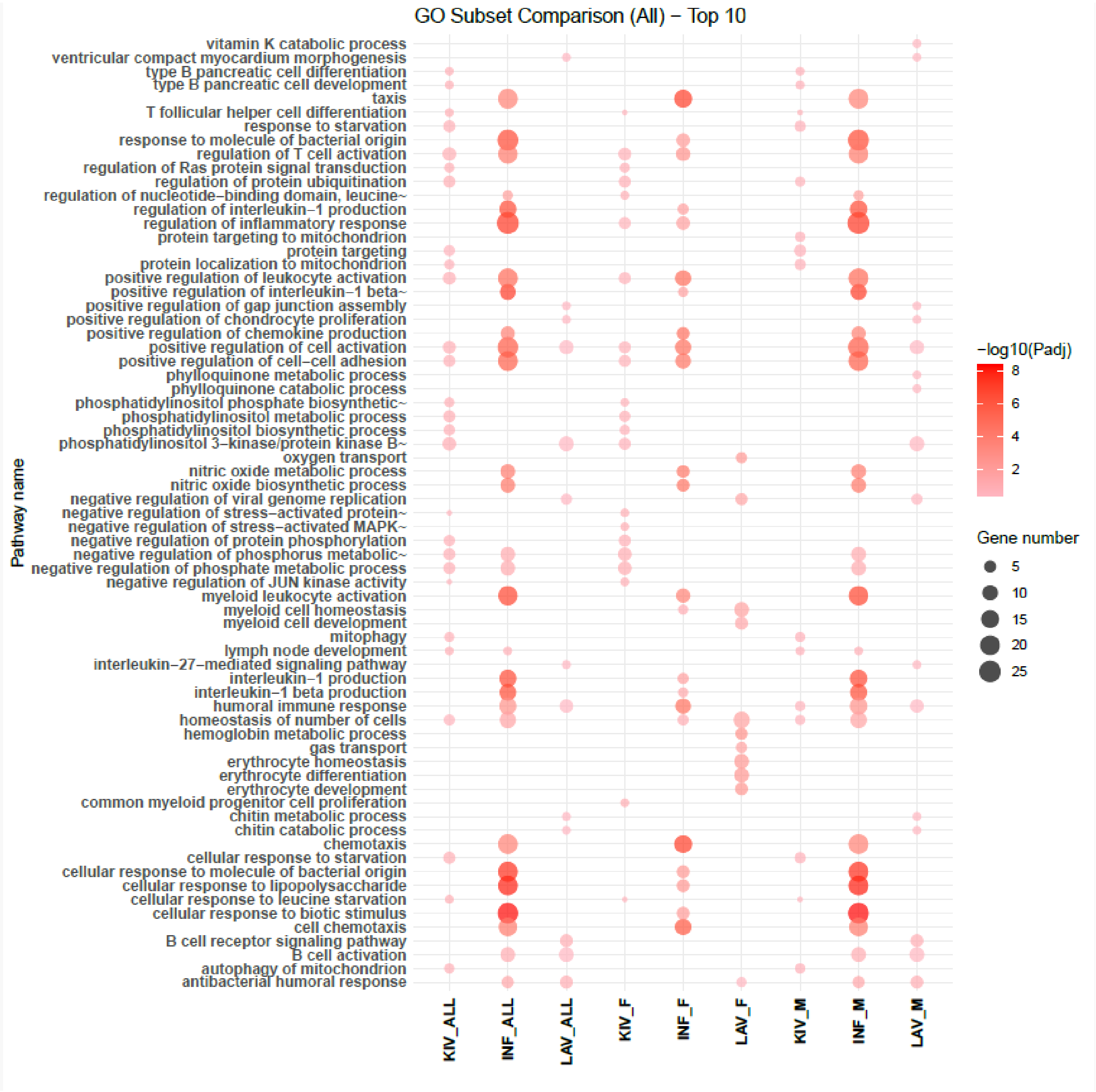

Supplemental Figure 11. GO functional analysis results of influenza vaccines. Any pathway listed shows up as one of the top-10 most significant GO pathways for one of the nine gene sets.

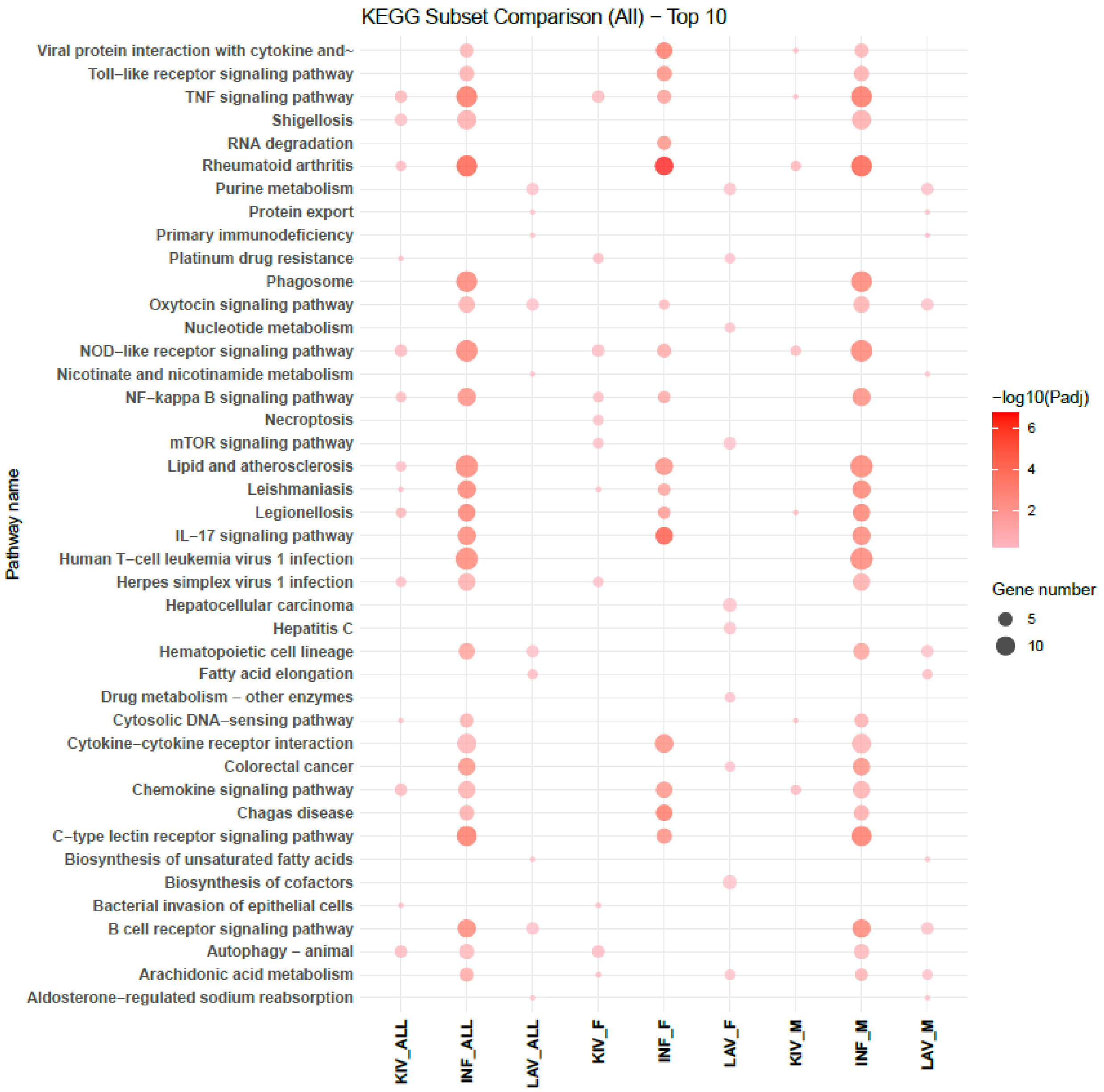

Supplemental Figure 12. KEGG functional analysis results of influenza vaccines. Any pathway listed shows up as one of the top-10 most significant KEGG pathways for one of the nine gene sets.

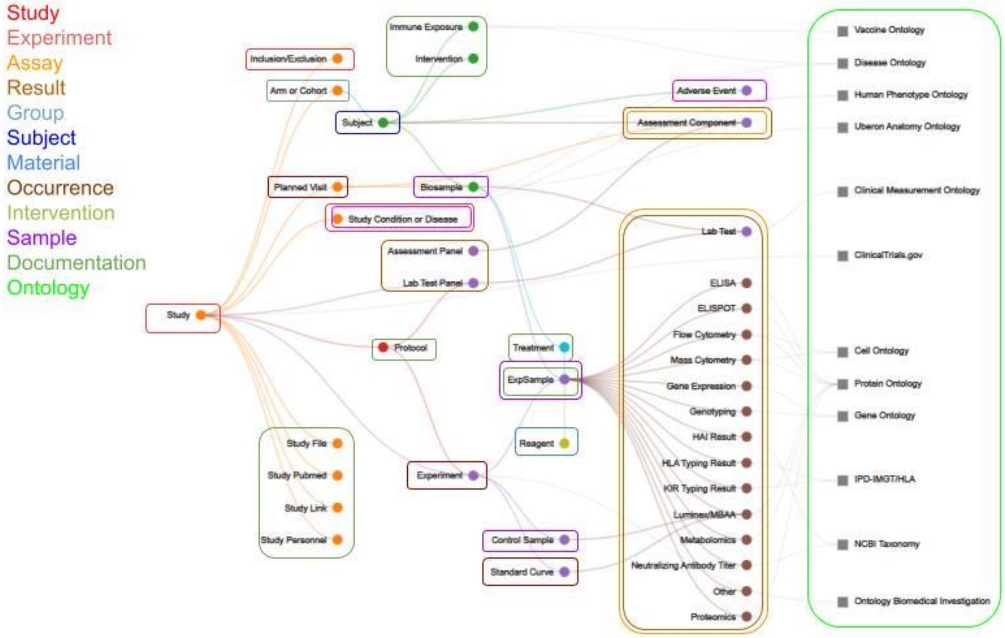

Supplemental Figure 13. ImmPort to SEA-CDM format modeling. A simplified mapping of key tables of ImmPort to SEA-CDM format. SEA-CDM foreign ids require information that are found in linking tables to consolidate data (i.e. SEA-CDM Sample requires data loaded from the “Biosample”, “ControlSample”, “ExpSample” core tables and “Biosample-2-Expsample”. Additionally, information related to SEA-CDM Sample’s Subject would require use of the “Biosample-2-Subject” table. The original connections showing each table was taken from the ImmPort website.
