# Supplementary material for: SEA CDM: Study-Experiment-Assay Common Data Model and Databases for Cross-Domain Data Integration and Analysis
Source: Sci Data. 2026 Jan 14;13:238. doi: 10.1038/s41597-026-06558-z (PMC12905146; doi:10.1038/s41597-026-06558-z)
Supplement: Supplementary file 3 — Supplemental File 2 [file 41597_2026_6558_MOESM3_ESM.pdf]

This contains instructions to convert data into VIGET to SEA-CDM format.

Download the VIGET_metadata file 'immport_vaccine_expression_matrix_mapped_merged_approved_genes_091421.csv', from Zenodo https://zenodo.org/records/7407195. If accessing this from Scientific data, download the 'OSEAN-VIGET-DB-Dumpfile.zip' from Zenodo https://zenodo.org/records/17770032.

Run ‘Osean_Viget_ETL_T.py’ using the path to 'immport_vaccine_expression_matrix_mapped_merged_approved_genes_091421.csv' as an input.

This will allow you to fill out the VIGET database.

Otherwise, you can generate the files using the ‘SEA-CDM_Final.zip’.

Use ‘SEA-CDM.sql’ to load the schema for OSEAN-DB.

Use the ‘OSEAN_db.schema.mwb ‘to load data into OSEAN-DB.

If you just want to use SEA-CDM files, fill out ‘SESA-CDM_template.zip’.

Please access our GitHub to see other ETLs for different databases (https://github.com/sea-cdm/OSEAN-DB) or recreate OSEAN-KG (https://github.com/sea-cdm/OSEAN-KG).
